# Radium tracing nutrient inputs through submarine groundwater discharge in the global ocean

**DOI:** 10.1038/s41598-018-20806-2

**Published:** 2018-02-05

**Authors:** Hyung-Mi Cho, Guebuem Kim, Eun Young Kwon, Nils Moosdorf, Jordi Garcia-Orellana, Isaac R. Santos

**Affiliations:** 1 School of Earth and Environmental Sciences/RIO, Seoul National University, 1 Gwanak-ro, Gwanak-gu, Seoul, 08826 Korea; 2 IBS Center for Climate Physics, Pusan National University, 2 Busandaehak-ro 63beon-gil, Geumjeong-gu, Busan, 46241 Korea; 3 Leibniz Center for Tropical Marine Ecology, Fahrenheitsrasse 6, 28359 Bremen, Germany; 4 Department de Física - Institut de Ciència i Tecnologia Ambientals, Universitat Autònoma de Barcelona, E-08193 Bellaterra, Catalonia Spain; 5 National Marine Science Centre, Southern Cross University, PO Box 4321, Coffs Harbour, 2450 NSW Australia

## Abstract

Riverine and atmospheric inputs are often considered as the main terrestrial sources of dissolved inorganic nitrogen (DIN), phosphorus (DIP), and silicon (DSi) in the ocean. However, the fluxes of nutrients via submarine groundwater discharge (SGD) often exceed riverine inputs in different local and regional scale settings. In this study, we provide a first approximation of global nutrient fluxes to the ocean via total SGD, including pore water fluxes, by combining a global compilation of nutrient concentrations in groundwater and the SGD-derived ^228^Ra fluxes. In order to avoid overestimations in calculating SGD-derived nutrient fluxes, the endmember value of nutrients in global groundwater was chosen from saline groundwater samples (salinity >10) which showed relatively lower values over all regions. The results show that the total SGD-derived fluxes of DIN, DIP, and DSi could be approximately 1.4-, 1.6-, and 0.7-fold of the river fluxes to the global ocean (Indo-Pacific and Atlantic Oceans), respectively. Although significant portions of these SGD-derived nutrient fluxes are thought to be recycled within sediment-aquifer systems over various timescales, SGD-derived nutrient fluxes should be included in the global ocean budget in order to better understand dynamic interactions at the land-ocean interface.

## Introduction

**Figure 1:**
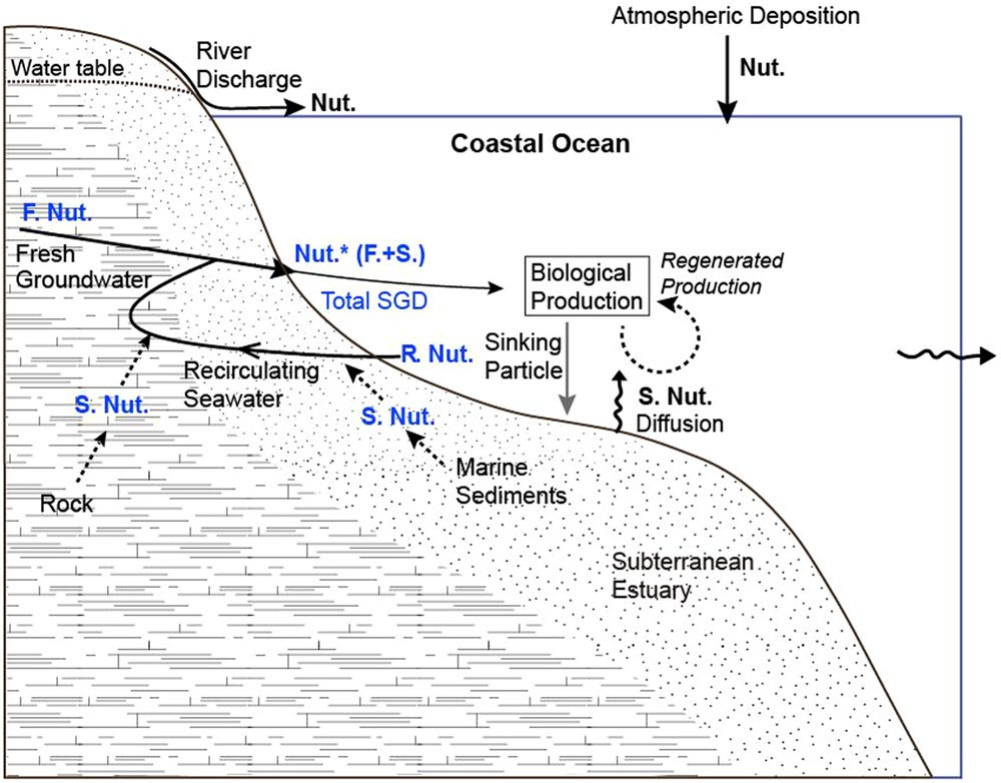
A schematic of biological production in the euphotic zone supported by nutrient inputs from atmosphere, rivers, and groundwater in the coastal ocean. F. Nut., R. Nut., and S. Nut. denote the nutrient sources from meteoric groundwater, recirculating seawater, and sediments and rocks, respectively. Nut.* denotes total nutrient inputs via SGD to coastal waters. The total flux includes the nutrients in fresh and salty groundwater resulting from reactions with rocks, sediments, and organic matter, except the nutrients included in seawater recirculating. In the coastal ocean, the diffusive fluxes of nutrients from bottom sediments in the euphotic zone are included in regenerated nutrients.

In the coastal ocean, nutrients could be supplied by advective inputs from aquifers and pore water, in addition to atmospheric and riverine inputs (Fig. 1). The advective fluxes may include nutrients from terrestrial sources and the remineralization of organic matter within the sediments (Fig. 1). In this study, we define submarine groundwater discharge (SGD) as any water advection into the ocean through the submarine ocean boundaries, which includes the discharge of fresh and saline groundwater as well as pore water advection^1–3^.

While global estimates are now available on the fluxes of nutrients to the global ocean via rivers and atmospheric deposition, no estimates are available for the SGD contribution to nutrient cycles in the global ocean. The total SGD flux has been revealed to be comparable to the river discharge to the Atlantic^4^ and global oceans^5,6^ using ^228^Ra (half life: 5.75 years) as a tracer. Several local studies have suggested that saline groundwater often delivers larger fluxes of solutes to the ocean than fresh groundwater^7–10^. From a basin scale perspective, nutrient inputs through total SGD were found to be comparable to those via rivers in the Mediterranean Sea^11^ and Yellow Sea^12^. Although saline groundwater is the dominant component of SGD globally^1,7^, estimates only exist for the contributions of “fresh” groundwater to dissolved inorganic nitrogen (DIN)^13^ and dissolved silicate (DSi)^14,15^ fluxes.

Here, we provide a first approximation of total SGD-derived nutrient fluxes to the global ocean using a simple mass balance approach. The main challenge to assess this flux is to determine a globally-significant groundwater endmember concentration for estimating SGD-derived nutrient fluxes. To do this, we combine a global compilation of nutrient data with the observationally constrained ^228^Ra flux estimate through SGD^5^ and compare the estimated fluxes with the better quantified river sources.

## Results and Discussion

### The global groundwater endmember

**Figure 2:**
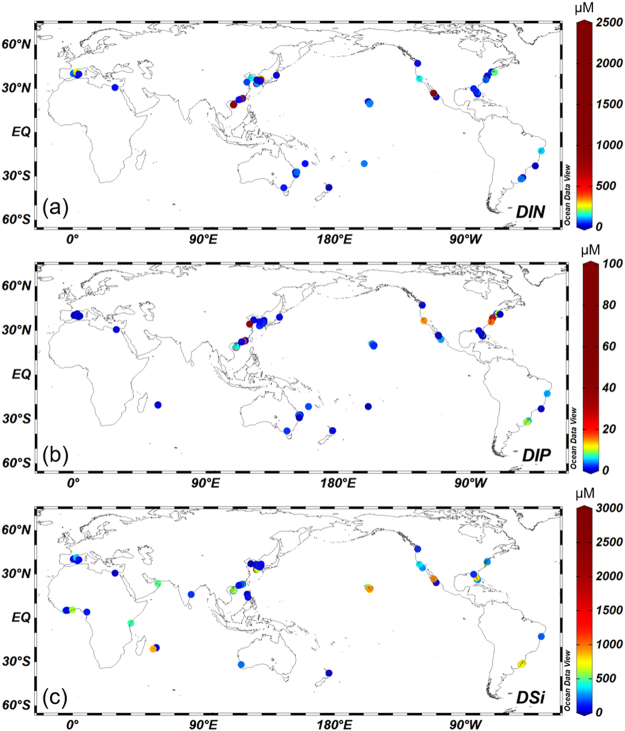
The distributions of data collection sites and concentrations for (**a**) DIN (n = 966), (**b**) DIP (n = 1001), and (**c**) DSi (n = 784), respectively, in world-wide coastal groundwater. The plots were created using Ocean Data View software version 4.7.6. (http://odv.awi.de).

**Figure 3:**
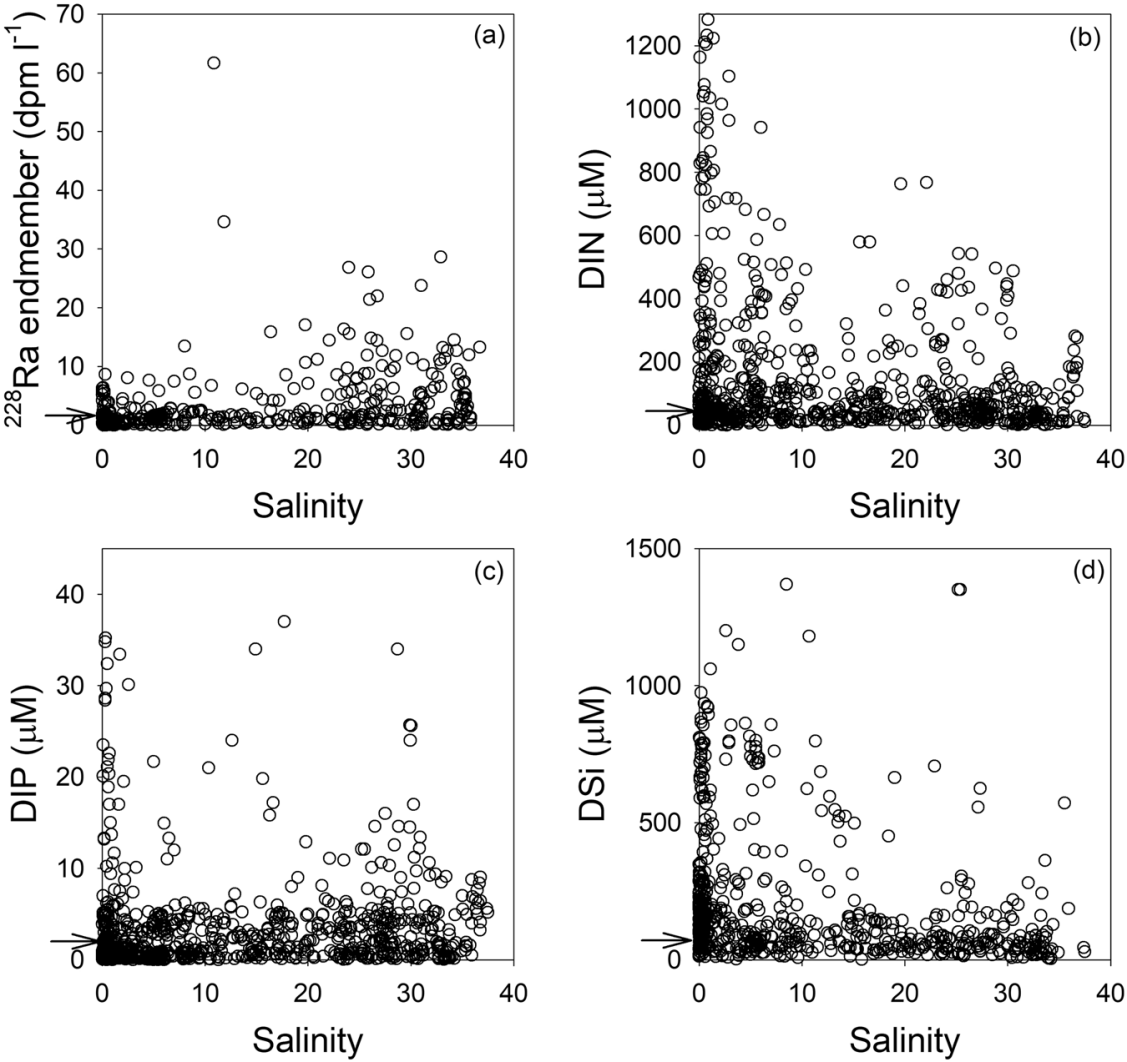
Plots of the concentrations of (**a**) ^228^Ra, (**b**) DIN, (**c**) DIP, and (**d**) DSi versus the salinities of groundwater samples from world-wide coastal aquifers. The arrows denote the endmember values.

**Figure 4:**
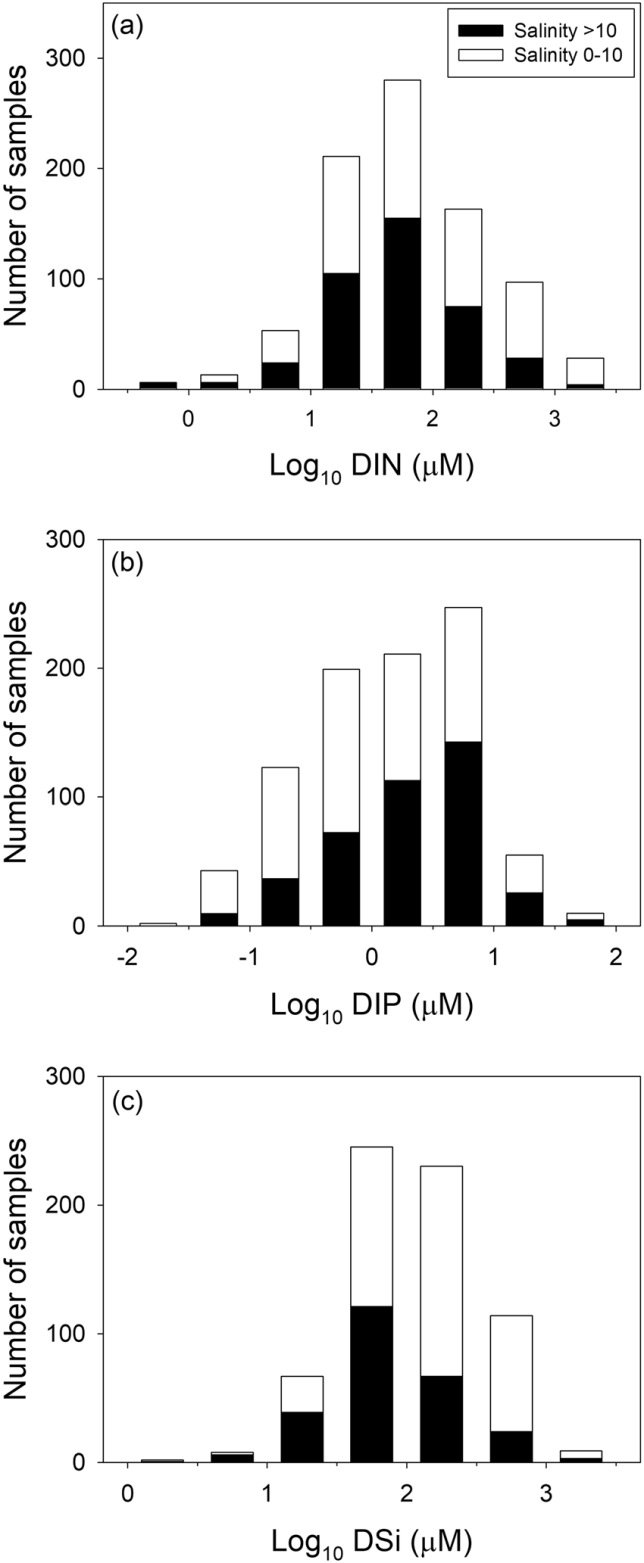
The histograms represent the distributions of (**a**) DIN, (**b**) DIP, and (**c**) DSi in coastal groundwater of the global ocean. The white and black bars represent salinity values lower and higher than 10, respectively.

Data for DIN (n = 966), dissolved inorganic phosphorus (DIP, n = 1001), DSi (n = 784), and ^228^Ra (n = 552)^6^ for global coastal groundwater (Fig. 2) and seawater were compiled (see Supplementary Note and Fig. S1). Approximately 800 sampling sites for groundwater nutrients (*Nutrient*_*gw*_) clustered along the east coasts of Asia and North America as well as the Mediterranean Sea, with scarce data elsewhere (Fig. 2). This clustering can affect the endmember concentrations of nutrients in groundwater. Therefore, a gridding method was used to examine the effect of the heterogeneity of geographical data distributions on determining the nutrient endmember values in groundwater. This method divides the globe into the horizontal resolution of 2° × 2° grid points, as examined previously for ^228^Ra^6^. All of the data within each grid point were averaged to represent the mean value at each grid point, assuming lognormal distributions of the tracer (see for example Figs 3 and 4 in Kwon, *et al*.^5^). No discernible difference was observed between the gridded global mean endmember and a bulk average without gridding (mean of the lognormal distribution; Supplementary Note and Fig. S2). Thus, in this study, the global mean groundwater endmember obtained by the mean of the lognormal distribution without gridding was used for the calculation of SGD-derived nutrient fluxes.

The sampling sites for the compiled *Nutrient*_*gw*_ data cover a wide range of environments including beach porewater, seepage zone, and inland wells near marginal seas, corresponding to a wide range of salinity (Fig. 3). The concentrations of DIN and DSi are much higher in fresh groundwater (salinity < 10) relative to saline groundwater (Supplementary Fig. S3). The higher DIN concentrations in lower salinity groundwater (Supplementary Fig. S3) may be associated with anthropogenic inputs, such as waste materials, fertilizers, manure, etc^16,17^. However, the higher concentrations of DSi in lower salinity groundwater (Supplementary Fig. S3) are more likely due to natural processes such as rock weathering and the dissolution of biogenic silica in sediments^18^. In contrast, the activity of ^228^Ra is much higher in saline groundwater (salinity >10; Supplementary Fig. S3) as explained by Cho and Kim^6^. The concentrations of DIP are highest and relatively constant in groundwater with salinity between 10 and 30 (Supplementary Fig. S3). The difference in DIP concentrations for different salinity ranges is unclear because it is affected by various factors: (1) groundwater contamination with DIP, (2) effects of DIP adsorption-desorption equilibria at the ambient salinity, (3) DIP remineralization by decomposing organic matter in coastal aquifers. Overall, the compiled data show that nutrient concentrations are remarkably constant at salinity values over 10 (Supplementary Fig. S3 and Table S1).

In the subterranean estuary, mixing of recirculating seawater with fresh groundwater creates an active biogeochemical reaction zone^19^. Thus, this zone significantly influences the fate of nutrients in the course of terrestrial nutrient transport from coastal aquifers to the ocean^20^. For example, redox conditions can affect the transformation processes and mobility of nitrogen (N) and phosphorus (P) in coastal aquifers^21^. Although these transformation processes influence most of the SGD-derived nutrient flux estimations so far reported in SGD studies^11,22–25^, the impact is still poorly understood. Further, biogeochemical conditions for the compiled nutrient samples are not well documented. Thus, instead of taking into account regional environmental conditions, we sort the nutrient samples according to the corresponding salinity values assuming that the groundwater salinity provides a first order criteria by which each nutrient sample can represent the globally averaged groundwater end member discharged into the marginal seas. As a first-order estimate of the global SGD endmember, we excluded fresh groundwater (salinity < 10) from the dataset (Fig. 4) under consideration, the approach taken by Cho and Kim^6^. For the chosen salinity range (>10), coastal aquifers may be less affected by biogeochemical transformation and anthropogenic contamination. Indeed, consistent values of our compiled data from global aquifers for this salinity range indicate that nutrients in this zone are mostly in equilibrium between sediments and pore water. This choice is also supported by the fact that fresh groundwater is a minor fraction of SGD (<10%)^1,7^, although several local studies have shown that fresh groundwater can seep directly in the mixing zone especially in porous volcanic and oceanic island environments^26–29^ and also karstic environments^30^.

### Calculation of the SGD-derived nutrient fluxes

The total SGD-derived nutrient fluxes (mol yr^−1^) are calculated using equation (1) (see also Supplementary Note)

1 $$\frac{Nutrien{t}_{gw}-Nutrien{t}_{sw}}{{}^{228}R{a}_{gw}-{}^{228}R{a}_{sw}}\times {}^{228}Ra\,flux=Total\,Nutrient\,Flux$$

where *Nutrient*_*gw*_ (mol m^−3^) and ^*228*^*Ra*_*gw*_ (dpm m^−3^) are the concentrations of DIN, DIP, and DSi and the endmember value of ^228^Ra in coastal groundwater aquifers, respectively. ^*228*^*Ra*_*gw*_ is obtained from Cho and Kim^6^. *Nutrient*_*sw*_ and ^*228*^*Ra*_*sw*_ are the concentrations of nutrients and ^228^Ra in the coastal seawater, obtained from Garcia *et al*.^31^ (Supporting Information Text S1 and Fig. S1) and Kwon, *et al*.^5^, respectively. The recirculated seawater component of SGD-derived nutrient and ^228^Ra fluxes are important, since we assume that the SGD fluxes are dominated by saline rather than fresh SGD^1,7^. ^228^*Ra flux* (dpm yr^−1^) is the flux of ^228^Ra into the global ocean through SGD, which was estimated to be 1.3 ± 0.3 × 10^17^ dpm yr^−1^ using a numerical model combined with a global compilation of ^228^Ra observations^5^.

### Global total nutrient fluxes

Our calculations show that total SGD-derived DIN, DIP, and DSi fluxes into the global ocean of 2.3 ± 0.6 Tmol yr^−1^, 0.06 ± 0.02 Tmol yr^−1^, and 3.8 ± 1.0 Tmol yr^−1^, respectively. The uncertainty represents one standard error from the average. These estimated SGD nutrient fluxes represent the total advective transport from continental margins to the coastal oceans through aquifers and pore waters. A significant fraction of the DIP and DIN in SGD may originate from the remineralization of organic matter within the sediments as observed in regional investigations^32,33^. Therefore, only small fractions of the total estimated SGD-derived DIP and DIN fluxes may be contributed by the net terrestrial fluxes to the ocean. However, a large portion of the DSi in SGD is likely to be released from aquifer solids^34^.

**Figure 5:**
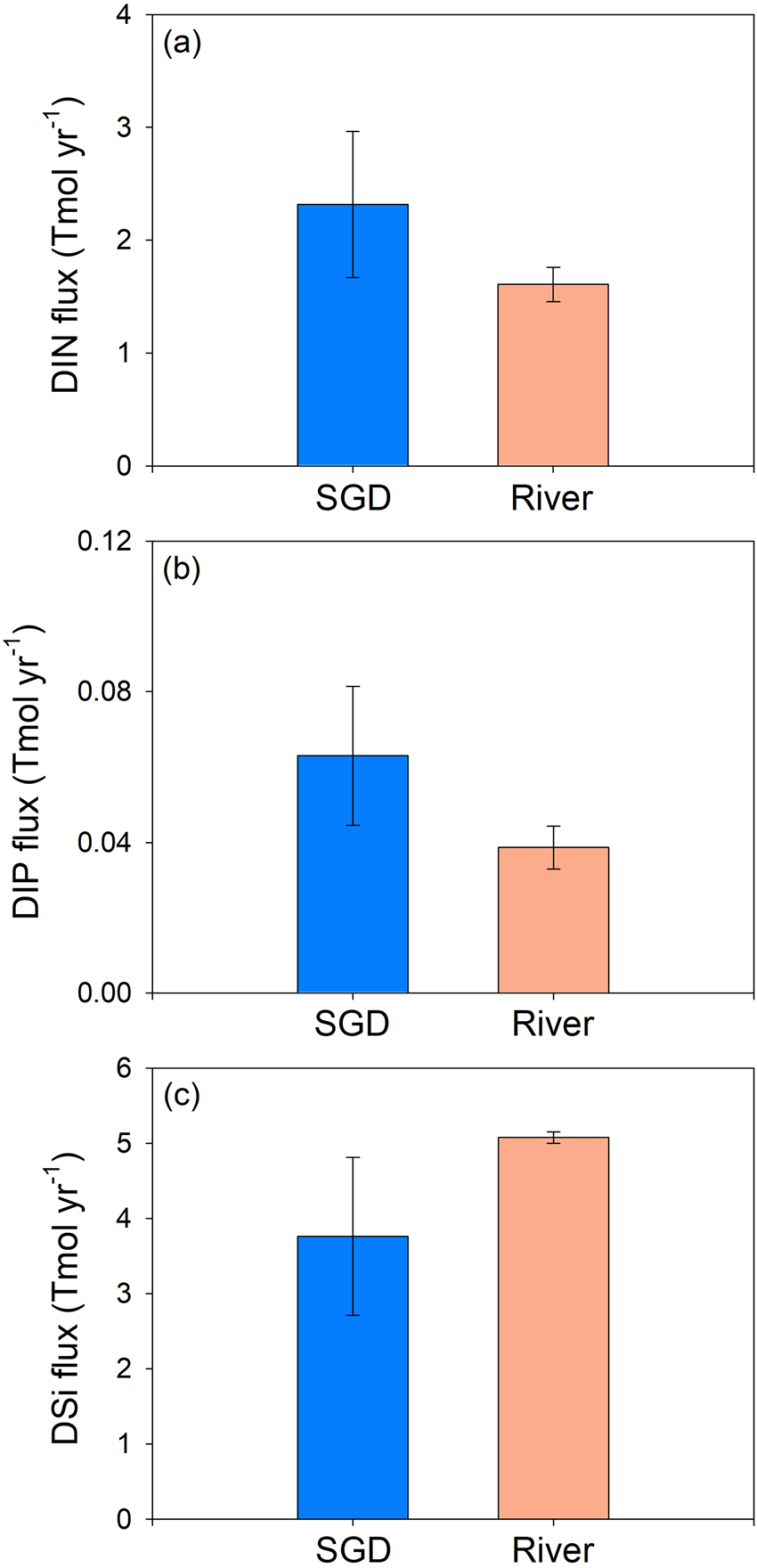
A comparison of global inputs of (**a**) DIN, (**b**) DIP, and (**c**) DSi to the ocean via SGD and river discharge. Fluxes via riverine inputs were obtained from the literatures^35,36^.

Our first order estimates provide an opportunity to compare SGD and river nutrient inputs to the ocean on a global scale for the first time, building on previous regional and local investigations. The estimated nutrient inputs through river discharge are from the Global Nutrient Export from Watersheds (Global NEWS) model, which includes river-basin-scale models that can predict export of dissolved and particulate nutrients using a function of natural and anthropogenic biogeophysical properties of 5761 exoreic basins^35,36^. The fluxes of riverine DIN, DIP, and DSi are approximately 1.6 ± 0.2 Tmol yr^−1^, 0.04 ± 0.006 Tmol yr^−1^, and 5.1 ± 0.1 Tmol yr^−1^, respectively^35,36^. Therefore, total SGD-derived fluxes of DIN, DIP, and DSi are approximately 1.4-, 1.6-, and 0.7-fold of the riverine fluxes to the global ocean, respectively (Fig. 5). Assuming that the nutrients supplied by total SGD are fully utilized by biological production, production supported by the total SGD-derived DIN flux (2.3 Tmol yr^−1^) could be potentially 15 Tmol C yr^−1^. These values are likely an underestimate of the actual fluxes since we use lower nutrient endmember ranges and higher Ra endmember ranges (data for salinity >10). Dissolved organic nitrogen and phosphorus would also increase the contribution of SGD to marine nutrient budgets^33,37^.

Estimates of nutrient fluxes from fresh groundwater have been attempted in previous studies^13–15^. Based on our compiled dataset, combining the globally averaged fresh SGD^21,38^ of 1.85 Tm^3^ yr^−1^ with the endmember value of DIN in fresh groundwater (salinity = 0; 56 ± 23 μM), the resulting DIN flux from fresh component of SGD is 0.10 ± 0.04 Tmol yr^−1^. This value agrees with the N input to the global ocean estimated by a land- and process-based modeling study (0.1 Tmol yr^−1^)^13^. Using the same method, the DIP (salinity = 0; endmember value of DIP = 0.6 ± 0.2 μM) and DSi (salinity = 0; endmember value of DSi = 131 ± 18 μM) fluxes via fresh-SGD are estimated to be 0.0012 ± 0.0004 Tmol yr^−1^ and 0.24 ± 0.03 Tmol yr^−1^, respectively. The DIP flux through fresh SGD is reported here for the first time. The DSi flux estimated in this study is approximately 30% of that reported by upscaling results^14^ from the two regional studies, which used an average groundwater DSi value of 340 μM from southern Brazil^39^ and Bengal Basin^40^. However, our estimated global DSi endmember value in coastal groundwater (salinity = 0) is approximately 130 μM, which is much lower than the value in the previous studies. The general consistency with the previous estimates for the fresh-SGD provides confidence that our globally compiled nutrient dataset is reasonable even though it assumes no biogeochemical transformations within coastal aquifers.

Our results suggest that saline groundwater plays a dominant role in the SGD-derived nutrient fluxes to the coastal ocean and that the SGD nutrients so far reported^13–15^ based on fresh groundwater discharge significantly underestimate the actual fluxes by total SGD. As is the case for the recirculating saline groundwater, large fractions of the SGD-derived nutrient fluxes are thought to be recycled through food webs in costal marine ecosystems and sedimentation processes (Fig. 1). This recycling can occur on various timescales ranging from hours to geological timescales associated with tides and long-term changes in sea levels, and hence has important implications for the marine biogeochemical responses to environmental and climate changes. The combined effects of riverine, atmospheric, and SGD-derived nutrient inputs are crucial for sustaining marine productivity in marginal seas and possibly in the open oceans. A full understanding of the dynamic interactions among lands, marginal seas, and the open ocean and their responses to human activities can only be achieved by including SGD fluxes to local, regional, and global budgets.

## Electronic supplementary material

Dataset 1
